# Development and Psychometric Testing of the Self-Care in COVID-19 (SCOVID) Scale, an Instrument for Measuring Self-Care in the COVID-19 Pandemic

**DOI:** 10.3390/ijerph17217834

**Published:** 2020-10-26

**Authors:** Maddalena De Maria, Federico Ferro, Davide Ausili, Rosaria Alvaro, Maria Grazia De Marinis, Stefania Di Mauro, Maria Matarese, Ercole Vellone

**Affiliations:** 1 Department of Biomedicine and Prevention, Faculty of Medicine and Surgery, University of Rome Tor Vergata, Via Montpellier, 1, 00133 Rome, Italy; federico.ferro@students.uniroma2.eu (F.F.); rosaria.alvaro@uniroma2.it (R.A.); ercole.vellone@uniroma2.it (E.V.); 2 Department of Medicine and Surgery, Faculty of Medicine and Surgery, University of Milan-Bicocca, Via Cadore 48, 20900 Monza, Italy; davide.ausili@unimib.it (D.A.); stefania.dimauro@unimib.it (S.D.M.); 3 Research Unit of Nursing Science, Faculty of Medicine and Surgery, Campus Bio-medico University of Rome, Via Alvaro del Portillo, 21 00128 Rome, Italy; m.demarinis@unicampus.it (M.G.D.M.); m.matarese@unicampus.it (M.M.)

**Keywords:** self-care, COVID-19, infectious diseases, psychometrics, validity, reliability

## Abstract

Aim: To develop the Self-Care in COVID-19 (SCOVID) scale and to test its psychometric characteristics in the general population. Methods: We tested SCOVID scale content validity with 19 experts. For factorial and construct validity, reliability, and measurement error, we administered the 20-item SCOVID scale to a sample of 461 Italians in May/June 2020 (mean age: 48.8, SD ± 15.8). Results: SCOVID scale item content validity ranged between 0.85–1.00, and the total scale content validity was 0.94. Confirmatory factor analysis supported SCOVID scale factorial validity (comparative fit index = 0.91; root mean square error of approximation = 0.05). Construct validity was supported by significant correlations with other instrument scores measuring self-efficacy, positivity, quality of life, anxiety, and depression. Reliability estimates were good with factor score determinacy, composite reliability, global reliability index, Cronbach’s alpha, and test-retest reliability ranging between 0.71–0.91. The standard error of measurement was adequate. Conclusions: The SCOVID scale is a new instrument measuring self-care in the COVID-19 pandemic with adequate validity and reliability. The SCOVID scale can be used in practice and research for assessing self-care in the COVID-19 pandemic to preventing COVID-19 infection and maintaining wellbeing in the general population.

## 1. Introduction

The COVID-19 pandemic, caused by the Sars-Cov-2 virus, has profoundly affected the lives of the global population. As of 18 October 2020, over 40 million people were affected by COVID-19, and 1100 million deaths were recorded worldwide [1]. According to the current evidence, the Sars-Cov-2 is transmitted by direct contact with infected individuals through respiratory droplets and by indirect contact through the transfer of the virus from contaminated hands or surfaces to the mouth, nose, or eyes [2,3]. Infected droplets can also be spread by asymptomatic subjects [3], making epidemic containment more challenging [4].

Several global health organizations (e.g., the World Health Organization [WHO], the American Center for Disease Control [CDC], the European Center for Disease Control [ECDC]) have recommended specific behaviors to reduce the contagion risk of Sars-Cov-2 in the population [5,6,7]. These behaviors (e.g., social distancing) are suggested to prevent the spread of COVID-19 [5,6,7] and to maintain individual wellbeing while coping with the distress caused by the pandemic (e.g., maintaining a daily routine and good sleep) [5,6,7].

Behaviors aimed at preventing disease and maintaining individual wellbeing are commonly defined as self-care behaviors [8]. For the purpose of this study, we developed the following definition of self-care in the COVID-19 pandemic: self-care is the decision-making process aimed at preventing COVID-19 and maintaining wellbeing in the COVID-19 pandemic. This definition is based on a prior theorization of self-care [8] and recommendations concerning the COVID-19 pandemic issued by national and international organizations [5,6]. Within the concept of self-care in the COVID-19 pandemic, we identified five dimensions based on the recommended behaviors: “individual protective measures”, “social distancing”, “environmental disinfection” pertaining to preventing COVID-19, “psychological wellbeing”, and “healthy lifestyle” pertaining to maintaining wellbeing. Evidence has shown that self-care is a key component in the prevention of diseases and in the maintenance of wellbeing in several conditions. For example, self-care behaviors such as social distancing and wearing a face mask have been associated with reducing the risk of contracting COVID-19 [9]. In many other conditions, better self-care has been found to be associated with better quality of life and better patient outcomes, including mortality [10,11].

Our definition of self-care inspired the development of the Self-Care in COVID-19 (SCOVID) scale, a new instrument that measures self-care behaviors preventing COVID-19 and maintaining wellbeing during the COVID-19 pandemic. As some studies have emphasized, COVID-19 has been challenging for individuals globally, not only for the prevention of the infection but also in maintaining their wellbeing, which could be threatened by the fear of contagion [12]. Thus, a valid and reliable instrument aimed at measuring self-care behaviors in the general population that could be implemented to prevent COVID-19 and to maintain wellbeing during the COVID-19 pandemic could be useful. This instrument would allow public healthcare professionals and researchers to assess the behaviors performed to prevent the risk of COVID-19 contagion and the alteration of wellbeing in the population. Therefore, the aim of this study was to develop a novel instrument, the SCOVID scale, and to test its psychometric properties of validity and reliability in the general population.

## 2. Materials and Methods

**Figure 1:**
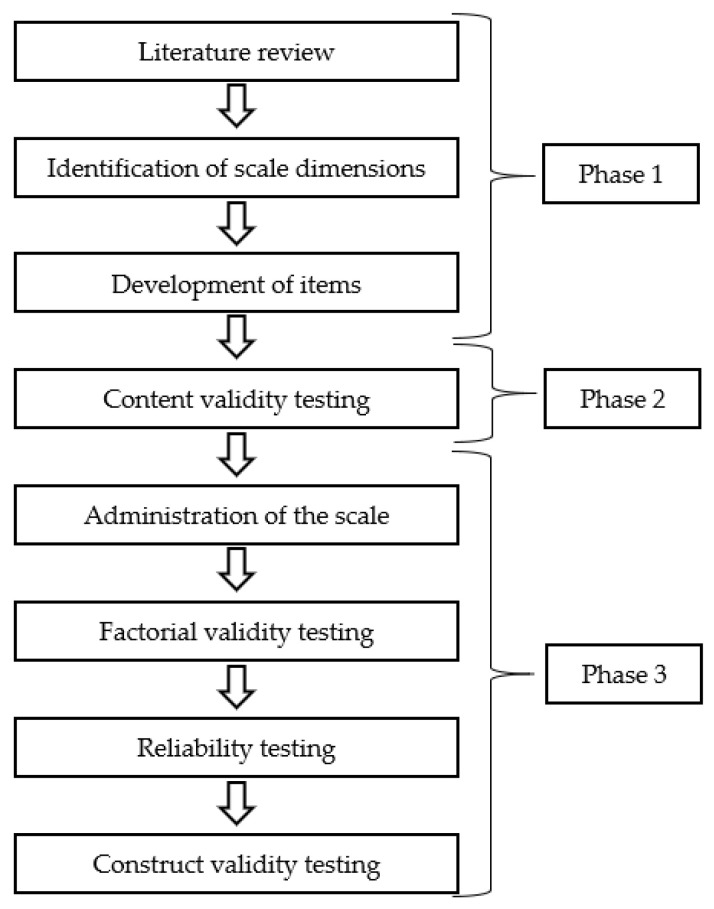
Workflow of the development of the Self-Care in COVID-19 (SCOVID) scale.

We developed and tested the SCOVID scale in a three-phase process, described in the following workflow (Figure 1).

(a) First Phase: Development of the SCOVID Scale Items:

In this phase, we reviewed the literature [13] and recommendations issued by national and international organizations (WHO, CDC, ECDC) addressed to the general population [5,6,7], and we identified 20 potential items complying with our definition of self-care in the COVID-19 pandemic. These items were grouped in the pre-identified self-care dimensions, as follows: “individual protective measures”, four items; “social distancing”, four items; “environmental disinfection”, three items; “psychological wellbeing”, five items; and “healthy lifestyle”, four items. To each item, we assigned a 5-point Likert scale for responses indicating the frequency of the behavior from 1 (never) to 5 (always).

(b) Second Phase: Content Validity of the SCOVID Scale Items:

For content validity, we followed the standards proposed by the COnsensus-based Standards for the selection of health Measurement INstruments (COSMIN) [14]. We selected a panel of 19 experts possessing expertise both in public health and in self-care. Through a Delphi survey, we asked experts to rate the relevance of each item in measuring the construct of self-care in the COVID-19 pandemic. Experts could rate each item on a 4-point Likert scale from 1 (“completely irrelevant”) to 4 (“completely relevant”). To assess comprehensiveness, we asked the experts to propose other items as well that they thought could describe remaining aspects of the construct of COVID-19 self-care not included in the items listed. After, we conducted cognitive interviews with a purposive sample of 20 individuals (mean age 47.3, SD ± 19.5, 60% were female, 55% had ≤8 years education) representing the general population to establish the comprehensibility of the items, instructions, and score response and to check if the intended meaning of each item was understood; further, we asked for suggestions regarding other behaviors performed by the participants to prevent COVID-19 and guarantee their wellbeing.

(c) Third Phase: Testing of the SCOVID Scale:

To test the scale factorial and construct validity and reliability, we administered the SCOVID scale to a quota sample representative of the general Italian adult population stratified by age and gender according to the Italian Institute of Statistics (ISTAT) in 2019. We adopted the following inclusion criteria to select participants for testing: (1) aged 18–80 years old; (2) comprehension of the Italian language, and: (3) living in Italy during the epidemic period. We excluded healthcare workers and people already infected by the Sars-Cov-2 from the study because the first sub-population is presumed to have more knowledge of COVID-19, and the second is more interested in performing behaviors to manage the disease. We administered the SCOVID scale in May/June 2020 during the so-called “phase two” of the Italian COVID-19 pandemic, which included the reopening of many commercial and job activities along with strong recommendations related to COVID-19 prevention (e.g., frequent hand hygiene). We collected data through an electronic questionnaire created with Google Modules^®^ (Google, LLC; Mountain View, CA, USA); about 10 min were required for administration. In case participants were not able to complete the Google Modules^®^, we administered the SCOVID scale by telephone in about a 15-minute call. For test-retest reliability, we administered the scale a second time after two weeks in a subsample of 100 participants [15].

Factorial and construct validity were assessed through confirmatory factor analysis (CFA) and hypothesis testing, respectively. Since literature reports that people performing better self-care have better self-efficacy [16], greater positivity [17] and quality of life [18], and lower anxiety [19] and depression [20], we administered the following instruments in association with the SCOVID scale for hypothesis testing. The *General Self-Efficacy* (GSE) *scale* [21] is an eight-item instrument that assesses self-efficacy in life in the general population. The total GSE scale score ranges from 1–5, with higher scores indicating higher self-efficacy. The *Positivity scale* [22] is an eight-item instrument that evaluates people’s positivity in life. The Positivity scale’s scores range between 1 and 5, with a higher score meaning higher positivity. The *Short Form 36 Health Survey (SF-36)* [23] is a 36-item generic instrument that evaluates quality of life in eight dimensions. Each dimension has a possible score of 0–100, with a higher score indicating a better quality of life. The *General Anxiety Disorder-7 (GAD-7)* [24] is a seven-item scale that assesses anxiety in the general population. Scores can range from 0–21, where higher scores designate greater anxiety. The *Patient Health Questionnaire-9 (PHQ-9)* [25] is a nine-item scale evaluating depressive disorders. The PHQ-9 total score ranges from 0–27, with a higher score indicating greater depression.

The SCOVID scale was administered within a study aimed at testing the psychometric characteristics of this scale in the general population during the COVID-19 pandemic. Participants were only asked to respond to the above instruments.

### 2.1. Ethical Considerations

The study was approved by an ethical committee of the University of Rome Tor Vergata (letter number: 157.20.) which is composed of an interdisciplinary team, including physicians from several specialties, nurses, lawyers, patient representatives, pharmacologists, a priest, and a statistician. Participants were fully informed about the study’s aims and gave their consent. Instruments were all anonymous, and patients were assured about data confidentiality. Also, participants were informed that they could withdraw from the study at any moment without giving a reason.

### 2.2. Data Analysis

The Content Validity Index (CVI) was used to assess the level of agreement among experts concerning each item’s relevance [26]. Experts’ responses of “1” (not relevant) and “2” (poor relevant) indicated poor validity and were coded as “0” while responses of “3” (relevant) and “4” (very relevant) were coded as “1” We calculated a mean score of all experts’ responses to obtain the CVI per each item (I-CVI), and we calculated the CVI for the entire SCOVID scale (S-CVI). A cut-off of ≥0.78 for I-CVI was used for item retention [26].

In the third phase, we performed data analyses in the following steps. In the first step, we used descriptive statistics to describe the sample sociodemographic characteristics and the responses to the SCOVID scale items. In the second step, we tested the SCOVID scale factorial validity. As the SCOVID scale was based on a theoretical definition of self-care, including five dimensions, we specified these dimensions in the CFA. Additionally, we specified the second-order factor “preventing COVID-19” to include “individual protective measures”, “social distancing”, and “environmental disinfection”. The second-order factor “maintaining wellbeing” included the “psychological wellbeing” and “healthy lifestyle” factors. Because the second-order factors are two dimensions of the entire SCOVID scale, we also specified a third-order factor to include the two second-order factors. As several items slightly violated the normality assumption, we used the robust maximum likelihood (MLR) method for parameter estimation. In the CFA, we used the following goodness-of-fit indices [27]: the Comparative Fit Index (CFI) and Tucker-Lewis Index (TLI), with values of 0.90–0.95 indicating acceptable fit, and values ≥ 0.95 indicating a good model fit; the Root Mean Square Error of Approximation (RMSEA), with values of ≤0.05 or 0.08 indicating a good fit as well as the rejection of the null hypothesis (for *p* < 0.05) associated with its 90% confidence interval; and the Standardized Root Mean Square Residual (SRMR), with values of 0.08 or less indicating a good fit. Moreover, we used traditional chi-square statistics, which were interpreted together with the above fit indices.

In the third step, we tested the construct validity via hypothesis testing by correlating the SCOVID scale scores with those of the other instruments. These correlations were performed with two-tailed Pearson’s *r*. A correlation coefficient ranging between 0.10–0.29 was considered weak, a coefficient ranging between 0.30–0.50 was considered moderate, and a coefficient >0.50 was considered strong [28].

In the third step, we evaluated the SCOVID scale’s reliability. Specifically, we computed the factor score determinacy coefficient [29] and the composite reliability [30] for every single first-, second-, and third-order factor as well as the global reliability index for multidimensional scales [31] and Cronbach’s alpha for the overall SCOVID scale. All of these reliability estimates should have a value >0.70. Finally, we tested the SCOVID scale test-retest reliability with the intraclass correlation coefficient (ICC) [15]. An ICC value of 0.75 is considered to represent good reliability, and a value greater than 0.90 demonstrates excellent reliability [15].

Finally, to evaluate responsiveness to changes, a measure of instrument precision, we tested the SCOVID scale measurement error using the standard error of measurement (SEM) and the smallest detectable change (SDC). The formula that we used for the SEM was standard deviation (SD) × √(1 − reliability coefficient) [32], where the SD was the SD of the SCOVID scale score, and the reliability coefficient was the factor score determinacy coefficient. The SEM identifies a more precise instrument if its value is <SD/2. The SDC was computed with the following formula: 1.96 × √ 2 × SEM [33]. The smaller the SEM and the SDC, the more precise the instrument. Analyses were conducted using SPSS 26.0^®^ (IBM Corp., Armonk, NY, USA) and Mplus V8.2 (Muthén & Muthén: Los Angeles, CA, USA) [29] for the CFA. 

## 3. Results

### 3.1. SCOVID Scale Content Validity

In the Delphi survey, each of the 20 items of the SCOVID scale obtained an I-CVI between 0.85 and 1.00, and the S-CVI was 0.94, demonstrating optimal relevance. Experts did not suggest further items indicating good comprehensiveness of the SCOVID scale. Cognitive interviews revealed that all items were easily understood by participants confirming good comprehensibility of the scale. These results indicate the excellent content validity of the SCOVID scale.

### 3.2. Sociodemographic Characteristics of Participants Who Completed the SCOVID Scale

Table 1 shows the sociodemographic characteristics of participants who completed the SCOVID scale in the third phase of the study. In total, 461 people were recruited, with a mean age of 48.8 years (SD ± 15.8), mostly female (52.3%), married (63.1%), and possessing a high school diploma (48.2%).

### 3.3. Descriptive Analysis of the SCOVID Scale Items

Table 2 shows the descriptive statistics of the SCOVID scale items. The average scores of the items ranged between 3.42 and 4.77. The item with the highest score was #16, measuring the frequency of wearing a face mask. The item with the lowest score was #8, measuring the frequency of physical activity. Not all items were normally distributed, with several showing a skewness and kurtosis greater than 1 (Table 2). To facilitate comparison, each factor and total scale score were standardized to produce a score from 0–100, with a higher score meaning better self-care.

### 3.4. Factorial Validity of the SCOVID Scale 

CFA for factorial validity testing was conducted by specifying the following five first-order factors: “individual protective measures” (items #1, #5, #13, #15, and #16), “social distancing” (items #3, #6, #12, and #17), “environmental disinfection” (items #2, #7, and #20), “psychological wellbeing” (items #4, #9, #11, and #19), and “healthy lifestyle” (items #8, #10, #14, and #18). Additionally, consistent with our theoretical definition of self-care, we specified two second-order factors: “preventing COVID-19”—including the first-order factors “individual protective measures”, “social distancing”, and “environmental disinfection”—and “maintaining wellbeing”—including the first-order factors “psychological wellbeing” and “healthy lifestyle.” Additionally, we specified a third order-factor that included the two second-order factors. However, this model could not be identified because it showed a correlation greater than |1| between the “psychological wellbeing” and the “healthy lifestyle” factors. This was evidence that the two factors belonged to the same factor. Consequently, we specified the items of the above two factors in a new first-order factor that we called “maintaining wellbeing.”

**Figure 2:**
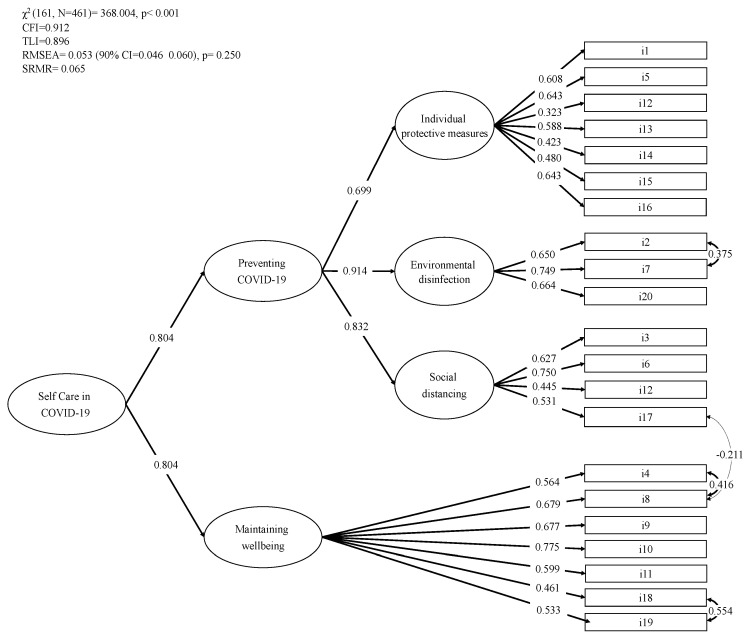
Confirmatory factor analysis of the SCOVID scale. *Note*: The results come from Mplus and are completely standardized solutions. All coefficients were statistically significant (*p* < 0.05). The numbers near the one-headed arrows are factor loading coefficients; the numbers near the two-headed arrows are correlation coefficients.

The specification of this new model with three first-order factors (“individual protective measures”, “social distancing”, and “environmental disinfection”) loaded by a second-order factor (“preventing COVID-19”) and the first-order factor “maintaining wellbeing” yielded inadequate but improvable fit indices: χ^2^(164, *N* = 461) = 577.504, *p* < 0.001, CFI 0.824, TLI = 0.796, RMSEA = 0.074 (90% CI = 0.067, 0.081), *p* < 0.001, SRMR = 0.070. Inspection of the modification indices revealed that the misfit was caused by item #14 (“maintaining a healthy life-style”), specified in the “maintaining wellbeing” factor, which was also loaded on “individual protective measures” and item #12 (“respect a distance of at least one meter from other people away from the home”), specified in the “social distancing” factor, which loaded on the “individual protective measures” factor. In addition, the modification indices showed an excessive covariance between residual of items #19 and #18, items #17 and #8, items #8 and #4, and items #2 and #7. The specification of the above residual covariances and the covariance between the “preventing COVID-19” and “maintaining wellbeing” factors fit the data well: χ^2^(161, *N* = 461) = 368.004, *p* < 0.001, CFI = 0.912, TLI = 0.896, RMSEA = 0.053 (90% CI = 0.046, 0.060), *p* = 0.250, SRMR = 0.065 (Figure 2). All factor loadings were statistically significant (*p* < 0.001).

### 3.5. Construct Validity of the SCOVID Scale

Table 3 shows the Pearson’s correlations between the SCOVID scale score and the other instruments’ scores. A positive strong correlation was found between the SCOVID scale and GSE (*r =* 0.503), as expected, while moderate and weak negative correlations were identified with the PHQ-9 (*r* = −0.330) and GAD-7 (*r* = −0.219), respectively. The SCOVID scale was also weakly positively correlated with all the SF-36 dimensions, except for the pain dimension, supporting the hypotheses formulated.

### 3.6. Reliability of the SCOVID Scale

The reliability estimates of the SCOVID scale are synthesized in Table 4. The reliability estimates were good with factor score determinacy, composite reliability, global reliability index, Cronbach’s alpha, and test-retest reliability ranging from 0.71 for the “environmental disinfection” factor to 0.91 for the “maintaining wellbeing” factor.

### 3.7. Measurement Errors of the SCOVID Scale 

The SEM of the SCOVID scale resulted in 4.54 for “individual protective measures”, 4.84 for “social distancing”, 6.96 for “environmental disinfection”, 5.46 for “maintaining wellbeing”, and 4.57 for the “preventing COVID-19” second-order factor. The SEM for the total SCOVID score resulted in 5.69. These measures were considered adequate. The SDC resulted in 12.58 for “individual protective measures”, 13.41 for “social distancing”, 19.29 for “environmental disinfection”, 15.13 for “maintaining wellbeing”, and 12.67 for the “preventing COVID-19” second-order factor. The SDC for the total SCOVID score resulted in 15.77.

## 4. Discussion

In this study, we developed and tested the SCOVID scale, a new instrument that measures self-care behaviors in the COVID-19 pandemic in the general population. We found that the SCOVID scale has good factorial and construct validity, reliability, and good precision. To our knowledge, this is the first instrument measuring self-care in the COVID-19 pandemic with important clinical and scientific implications.

The SCOVID scale underwent a rigorous process of development and testing. In the development process, we selected items from the literature and from the recommendations of national and international organizations. Consequently, the SCOVID scale items are based on the best available knowledge regarding the prevention of COVID-19 and the maintenance of wellbeing in the COVID-19 pandemic. Additionally, we based the SCOVID scale on a self-care definition rooted in existing self-care theories. Interestingly, the initial solution with five first-order factors could not be identified because the “psychological wellbeing” and “healthy lifestyle” items belonged to the same factor that we named “maintaining wellbeing”, as in our definition of self-care.

The initial model fit of the SCOVID scale was partially supported by the CFA and we had to specify another factor for items #12 and #14. Item #12 is related to respecting a distance of at least one meter from other people outside the home and was initially specified in the “social distancing” factor; item #14 measures the maintenance of correct life-styles and was initially specified in the “healthy lifestyle” factor. However, our analysis showed that both items #12 and #14 loaded better in the “individual protective measures” factor, showing that respecting a social distance and maintaining a healthy lifestyle were interpreted as individual protective measures by our participants. Moreover, we found correlations among some item residuals that could be expected (e.g., the correlations between the residuals of items #19, measuring the maintenance of a daily routine, and #18, measuring the sleep-wake rhythm, are both related to routine). According to the literature [34,35], it is acceptable to allow item residuals to correlate if there are theoretical and methodological justifications, as in our case.

Construct validity of the scale was supported by the evidence of correlations between the SCOVID scale scores and other instruments’ scores measuring related constructs. In fact, the literature reports that people performing better self-care have better outcomes in terms of quality of life and psychological wellbeing and people with higher self-efficacy perform better self-care [10,11].

The SCOVID scale reliability testing was supported by all our reliability estimates. The established reliability at the first, second, and third levels is important because it allows measurement with good reliability of the specific dimensions, as measured with first- and second-order factors, and of the entire scale score. Furthermore, SEM analysis could be considered an acceptable measurement error for the SCOVID scale score because the result was below half of the SD of each factor and for the overall scale. Regarding the SDC, we have been able to establish how many points in the SCOVID scale at factor and scale levels we may consider for a meaningful change. Since both SEM and SDC were small, we can assume that the scale is precise in the measurement of self-care in COVID-19 behaviors.

This study has several limitations. First, we collected data using a convenience sample. We tried to balance this limitation by enrolling a sample representative of the Italian population distributed by age and gender. Another limitation is that we did not collect data for people under 18 or over 80 years of age; consequently, our findings can be generalized with caution to these two subgroups of the population. Finally, a further limitation is that our results have been obtained from an Italian population that was particularly affected by COVID-19. Consequently, the generalization of our results to other countries with different spread of the infection should be done with caution. 

Our study has important public health implications. We demonstrated the real existence of self-care in the COVID-19 pandemic construct, which could favor the development of a more organized theory on this topic. From a clinical point of view, the SCOVID scale could be a valuable instrument to measure to what extent individuals adopt all self-care behaviors recommended in the COVID-19 pandemic. In case individuals have lower scores at the SCOVID scale, they could be at risk of poor self-care and clinicians could provide them with tailored interventions to improve their self-care behaviors. From a scientific point of view, the SCOVID scale would allow the identification of predictors and outcomes related to self-care in the COVID-19 pandemic, which have not yet been identified. Additionally, the COVID scale could be used in experimental studies to evaluate the effectiveness of interventions aimed at improving self-care. 

## 5. Conclusions

In this study, we developed the SCOVID scale and demonstrated that this instrument has good validity and reliability. We suggest its use in clinical practice to evaluate the self-care behaviors performed by individuals in the COVID-19 pandemic and in research to describe self-care in the COVID-19 pandemic with its predictors and outcomes. 

## Figures and Tables

**Table 1:** Sociodemographic characteristics of participants (*N* = 461).

Variable	*M* (*SD*)
Age	48.79 (15.81)
	*N* (%)
Gender	
Male	220 (47.7)
emale	241 (52.3)
Marital status	
Single	115 (24.9)
Married/Partnered	291 (63.1)
Divorced	33 (7.2)
Widowed	22 (4.8)
Level of education	
No title	2 (0.4)
Elementary school	24 (5.2)
Middle school	63 (13.7)
High school	222 (48.2)
University degree	150 (31.5)
Region of residence	
Northern Italy	45 (9.8)
Central Italy	200 (43.4)
Southern Italy	216 (46.9)
Nationality	
Italian	456 (98.9)
Other	5 (1.1)
Occupation	
Employee	156 (33.8)
Freelance	69 (15.0)
Worker	27 (5.9)
Student	31 (6.7)
Other	76 (16.5)
Unemployed	14 (3.0)
Retired	88 (19.1)
Perceived income adequacy	
Less than needed	15 (3.3)
Enough for living	307 (66.6)
More than needed	139 (30.2)

Legend. *M*: mean, *SD:* standard deviation.

**Table 2:** Descriptive statistics for individual items of the SCOVID scale (*N* = 471).

Item	*M*	*SD*	Skewness	Kurtosis
1. Wash your hands with water and soap or disinfectant solution after carrying out activities at risk of contagion (e.g., using public transport, grocery shopping in supermarkets)	4.74	0.59	−2.95	10.95
2. Ensure home hygiene using chlorine- or alcohol-based products	4.06	1.07	−1.10	0.48
3. Avoid handshakes and/or hugs with people other than members of your household	4.69	0.71	−2.93	9.86
4. Do something to relieve stress (e.g., take medication, do yoga, listen to music)	3.52	1.25	−0.43	−0.79
5. Don’t touch (your own) eyes, nose, or mouth with your hands when outside home, even when wearing gloves	4.28	0.98	−1.49	1.86
6. Avoid places where a distance of at least one meter between people is not respected	4.43	0.82	−1.57	2.65
7. Disinfect surfaces and objects shared with other people (e.g., handles, switches, desks, keyboards, remote controls, telephones)	3.82	1.24	−0.80	−0.39
8. Maintain regular physical activity (e.g., walking, running, exercise bike, online guided exercises)	3.42	1.30	−0.35	−0.95
9. Try to maintain your usual hobbies or cultivate new ones (e.g., reading, painting, gardening, cooking)	3.78	1.09	−0.72	0.00
10. Maintain a healthy and balanced diet appropriate to activities during the day	3.78	1.03	−0.71	0.18
11. Try to maintain a well-groomed appearance despite not being able to go to the hairdresser or beautician	4.01	0.97	−0.90	0.45
12. Maintain a distance of at least one meter from other people outside the home	4.64	0.65	−2.21	6.23
13. Wear disposable gloves in public places when there is a risk of contagion (e.g., on public transport, in the supermarket)	4.35	1.10	−1.79	2.34
14. Maintain a healthy lifestyle (e.g., avoiding or limiting smoking, not abusing alcohol or other drugs)	4.26	1.05	−1.47	1.42
15. Try to keep in touch with other people, other than members of your own household (e.g., friends, relatives, colleagues) by means of phone calls, video calls, e-mails, etc.	4.38	0.80	−1.36	2.07
16. Cover your nose and mouth (e.g., by using a face mask) when there is a risk of contagion	4.77	0.61	−3.33	12.93
17. Limit to leave home	4.23	0.95	−1.19	0.85
18. Maintain a regular sleep-wake rhythm (e.g., going to bed at the same time and waking up at the same time every day)	3.61	1.20	−0.51	−0.68
19. Try to maintain a daily routine	4.01	0.89	−0.68	0.12
20. Ensure air changes in rooms shared with other people (e.g., workplace, home)	4.57	0.66	−1.40	1.24
Total SCOVID scale score	79.19	13.40	−1.02	2.45
Individual protective measures score	87.21	13.73	−2.09	6.82
Environmental disinfection score	78.74	21.05	−1.01	0.31
Social distancing score	87.43	14.64	−1.73	4.04
Preventing COVID-19 score	85.03	13.16	−1.44	3.39
Maintaining wellbeing score	70.01	18.19	−0.48	0.29

Legend. *M:* mean, *SD*: standard deviation; *SCOVID*: Self-Care in COVID-19 scale.

**Table 3:** Pearson’s correlations between the SCOVID scale and other scales (construct validity) (*N* = 461).

SCOVID/Other Scales	General Self-Efficacy Scale	Positivity Scale	GAD-7 Scale	PHQ-9 Scale	SF-36							
					PF	PHL	EHL	E/F	EW	SF	P	GH
Individual protective measures	0.42 ^b^	0.39 ^b^	−0.16 ^b^	−0.28 ^b^	0.13 ^b^	0.23 ^b^	0.19 ^b^	0.20 ^b^	0.25 ^b^	0.24 ^b^	0.12 ^b^	0.20 ^b^
Environmental disinfection	0.32 ^b^	0.26 ^b^	−0.03	−0.14 ^b^	0.03	0.09	0.14 ^b^	0.12 ^b^	0.04	0.05	−0.12 ^b^	0.12 ^a^
Social distancing	0.3 ^b^	0.26 ^b^	−0.19 ^b^	−0.23 ^b^	−0.01	0.15 ^b^	0.16 ^b^	0.17 ^b^	0.14 ^b^	0.15 ^b^	−0.02	0.06
Preventing COVID-19	0.43 ^b^	0.38 ^b^	−0.15 ^b^	−0.26 ^b^	0.07	0.20 ^b^	0.20 ^b^	0.19 ^b^	0.18 ^b^	0.19 ^b^	0.00	0.16 ^b^
Maintaining wellbeing	0.48 ^b^	0.41 ^b^	−0.26 ^b^	−0.35 ^b^	0.16 ^b^	0.21 ^b^	0.22 ^b^	0.36 ^b^	0.25 ^b^	0.26 ^b^	0.04	0.24 ^b^
SCOVID Total	0.50 ^b^	0.44 ^b^	−0.22 ^b^	−0.33 ^b^	0.13 ^b^	0.22 ^b^	0.23 ^b^	0.30 ^b^	0.23 ^b^	0.24 ^b^	0.02	0.22 ^b^

Note: GAD-7: General Anxiety Disorder-7, PHQ-9: Patient Health Questionnaire-9, SF-36: Short form 36 health survey, PF: Physical Functioning, PHL: Physical Health Limitations, EHL: Emotional Health Limitations, E/F: Energy/Fatigue, EW: Emotional Wellbeing, SF: Social Functioning, P: Pain, GH: General Health; SCOVID: Self-Care in COVID-19 scale. Note: ^a^: *p* ≤ 0.05; ^b^: *p* ≤ 0.001.

**Table 4:** Reliability of the SCOVID scale.

SCOVID Factors and Scale	Factor Score Determinacy	Composite Reliability	Global Reliability Index	Cronbach’s Alpha	ICC (95% CI)
Individual protective measures	0.89	0.76			
Environmental disinfection	0.89	0.71			
Social distancing	0.89	0.73			
Preventing COVID-19	0.88				
Maintaining wellbeing	0.91	0.80			
SCOVID Total	0.82		0.91	0.88	0.91 (0.88–0.93)

Note: ICC = intraclass correlation coefficient; CI: confidence interval; SCOVID: Self-Care in COVID-19 scale.
